# Genomic Analysis of Undifferentiated Carcinoma of the Pancreas with Squamous Differentiation: A Case Report

**DOI:** 10.7759/cureus.55175

**Published:** 2024-02-28

**Authors:** Motoyasu Kan, Yusuke Kouchi, Hiroshi Ohyama, Genki Usui, Masaki Fukuyo, Shigetsugu Takano, Takashi Kishimoto, Atsushi Kaneda, Masayuki Ohtsuka, Naoya Kato

**Affiliations:** 1 Gastroenterology, Chiba University, Chiba, JPN; 2 Molecular Pathology, Chiba University, Chiba, JPN; 3 Molecular Oncology, Chiba University, Chiba, JPN; 4 General Surgery, Chiba University, Chiba, JPN

**Keywords:** swi/snf complex-related gene, chromatin remodeling, genomic analysis, undifferentiated carcinoma of the pancreas, pancreatic cancer

## Abstract

Pancreatic cancer is an intractable malignancy associated with a dismal prognosis. Undifferentiated carcinoma, a rare subtype, poses a clinical challenge owing to a limited understanding of its molecular characteristics. In this study, we conducted genomic analysis specifically on a case of undifferentiated carcinoma of the pancreas exhibiting squamous differentiation.

An 80-year-old male, previously treated for colorectal cancer, presented with a mass with central cystic degeneration in the pancreatic tail. The mass was diagnosed pathologically as undifferentiated carcinoma of the pancreas with squamous differentiation. Despite surgical resection and chemotherapy, the patient faced early postoperative recurrence, emphasizing the aggressive nature of this malignancy.

Genomic analysis of distinct histologic components revealed some common mutations between undifferentiated and squamous components, including Kirsten rat sarcoma virus (KRAS) and TP53. Notably, the squamous component harbored some specific mutations in SMARCA4 and SMARCB1 genes that code for members of the SWItch/Sucrose Non-Fermentable (SWI/SNF) chromatin remodeling complex.

The common mutations in the undifferentiated and squamous cell carcinoma components from this analysis suggest that they originate from a common origin. The discussion also underscores the scarcity of genomic analyses on undifferentiated carcinoma of the pancreas, with existing literature pointing to SWI/SNF complex-related gene mutations. However, our case introduces chromatin remodeling factor mutations as relevant in squamous differentiation.

In conclusion, this study provides valuable insights into the genomic landscape of undifferentiated pancreatic carcinoma with squamous differentiation. These findings suggest the importance of further research and targeted therapies to improve the management of undifferentiated carcinoma of the pancreas and enhance patient outcomes.

## Introduction

Pancreatic cancer, with a five-year survival rate of 12%, represents a challenging malignancy with poor prognosis [1]. Undifferentiated carcinoma, a rare histological subtype within pancreatic cancer, accounts for 2-3% of pancreatic tumors [2]. It is characterized by high cellularity, weak cell-to-cell adhesion, low stromal content, and a diverse population of cells with no clear differentiation direction. The World Health Organization classification includes undifferentiated carcinoma and undifferentiated carcinoma with osteoclast-like giant cells as subtypes of pancreatic ductal adenocarcinoma [3]. Undifferentiated carcinoma is further categorized into anaplastic undifferentiated carcinomas, sarcomatoid undifferentiated carcinomas, and carcinosarcomas. The prognosis of undifferentiated carcinoma is generally considered poor compared to conventional ductal adenocarcinoma, with a reported median survival of around four months in cases deemed unresectable [4].

Recent advances in cancer genomics have led to active exploration of the mechanisms of carcinogenesis and identification of therapeutic targets. While genomic analysis, transcriptome, and proteome integration have been reported for conventional ductal adenocarcinoma [5], there is insufficient consideration of genomic profiling in undifferentiated carcinoma of the pancreas. In this study, we report a case of undifferentiated carcinoma of the pancreas with squamous differentiation, where genomic profiling using target DNA sequences was performed.

This article was previously presented as a meeting abstract at the 54th Annual Meeting of the Japan Pancreas Society on July 21, 2023.

## Case presentation

**Figure 1:**
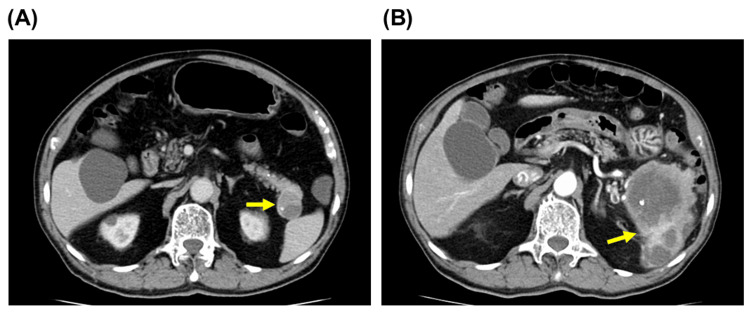
Contrast-enhanced CT images of the pancreatic tumor (A) Initial detection revealed a cystic lesion with calcification measuring 30 mm in the pancreatic tail (arrow). (B) At one year and eight months follow-up, significant enlargement and infiltration into the spleen were observed (arrow).

The patient was an 80-year-old man who had undergone curative resection by endoscopic submucosal dissection for early-stage colorectal cancer and was being followed up. A follow-up contrast-enhanced computed tomography scan revealed a 30 mm mass with central cystic degeneration in the pancreatic tail (Figure 1A). Although the mass was first suspected as a solid pseudopapillary neoplasm or involvement of an accessory spleen, the findings of diameter enlargement and splenic invasion were observed at one year and eight months after the initial discovery (Figure 1B). Distal pancreatectomy was performed, and the mass was diagnosed pathologically as undifferentiated carcinoma of the pancreas with squamous differentiation. 

Microscopically, the mass contained three histological components: (i) undifferentiated carcinoma (70%), (ii) squamous cell carcinoma (30%), and (iii) well to moderately differentiated conventional ductal adenocarcinoma (very focal). The histological findings of the undifferentiated carcinoma component were predominantly those of anaplastic giant cell carcinoma, with an extremely minor component of sarcomatoid carcinoma. Osteoclast-like giant cells were not observed throughout the lesion. Despite postoperative chemotherapy with S-1, the patient experienced difficulty in oral intake, suffered cardiac arrest during a hospital visit, and subsequently passed away two months after surgery. Pathological autopsy confirmed multiple liver metastases and peritoneal dissemination, attributing the cause of death to early postoperative recurrence.

**Figure 2:**
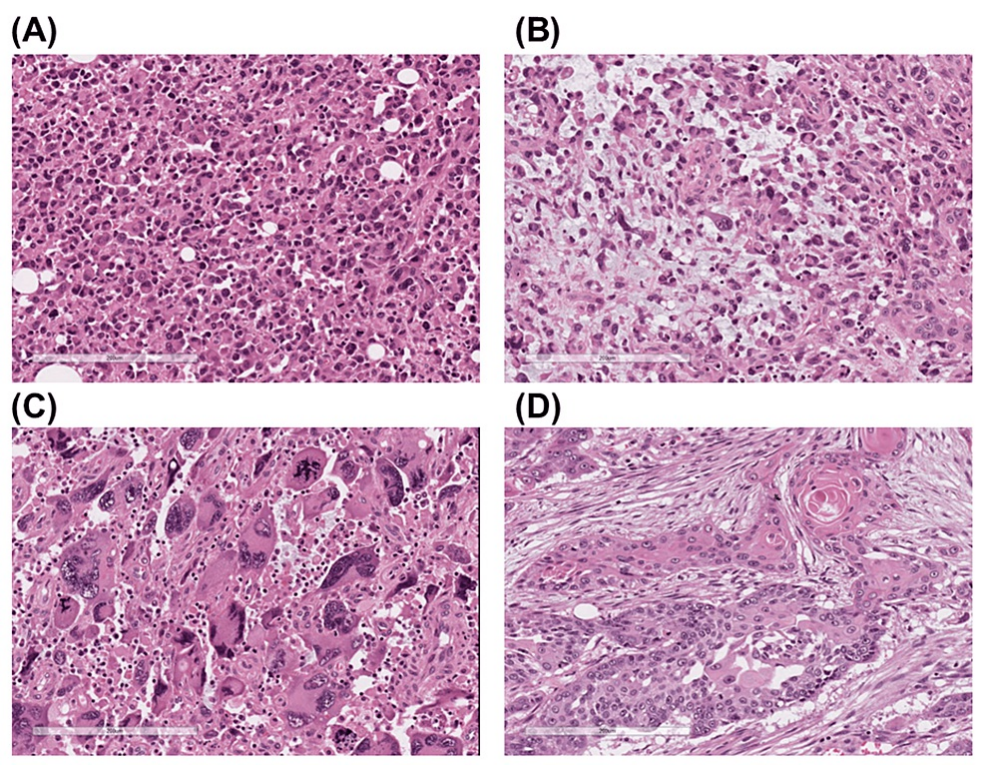
Histological findings of the resected specimen (A) Anaplastic undifferentiated carcinoma component with pleomorphic mononuclear cells. (B) Anaplastic undifferentiated carcinoma component with myxomatous stroma. (C) Anaplastic undifferentiated carcinoma component with neoplastic multinucleated giant cells. (D) Squamous cell carcinoma component with keratinization and intercellular bridges.

Formalin-fixed paraffin-embedded (FFPE) specimens from the distal pancreatectomy specimen were utilized for DNA extraction. Histologically, the following four distinct components were identified in each separate section: (A) anaplastic undifferentiated carcinoma component with poorly cohesive pleomorphic mononuclear cells, (B) anaplastic undifferentiated carcinoma component with myxomatous stroma, (C) anaplastic undifferentiated carcinoma component with neoplastic multinucleated giant cells, and (D) squamous cell carcinoma component (Figure 2). Macrodissection was performed, and DNA was extracted using the QIAamp DNA FFPE Tissue kit (Qiagen, Hilden, Germany). Subsequently, a quality check for DNA was conducted using the QIAseq DNA QuantiMIZE Assay Kit (Qiagen). Library preparation was carried out using QIAseq 12-Index I, and the quality of the library was assessed through the QIAseq library quant assay. Target DNA sequencing was performed using the Human Comprehensive Cancer Panel (Qiagen), capable of identifying single nucleotide variants, copy number variations, and small insertions and deletions. Gene mutations labeled as "likely oncogenic" and "oncogenic" according to OncoKB [6,7] were considered pathogenic.

Identified pathogenic mutations for each histological area are presented in Table 1. Common mutations in all four areas included KRAS (c.181C>A), TP53, FANCD2, TGFBR2, KDR, SUFU, ATM, AXIN1, and AURKA. Squamous cell carcinoma component harbored some distinct mutations in SMARCA4 and SMARCB1 genes that code for members of the SWItch/Sucrose Non-Fermentable (SWI/SNF) chromatin remodeling complex.

**Table 1:** Genomic analysis results DNA extracted from sections of the same surgical specimen exhibiting four different histologic types was analyzed. Common genetic mutations were identified among them. More gene mutations were identified in squamous cell carcinomas. The squamous cell carcinoma component exhibited a higher number of genetic mutations.

	Figure 2 (A)	Figure 2 (B)	Figure 2 (C)	Figure 2 (D)
KRAS	Missense_Mutation	Missense_Mutation	Missense_Mutation	Missense_Mutation
TP53	Missense_Mutation	Missense_Mutation	Missense_Mutation	Missense_Mutation
FANCD2	Splice_Region	Splice_Region	Splice_Region	Splice_Region
TGFBR2	Frame_Shift_Del	Frame_Shift_Del	Frame_Shift_Del	Frame_Shift_Del
KDR	Missense_Mutation	Missense_Mutation	Missense_Mutation	Missense_Mutation
KMT2C	Nonsense_Mutation Frame_Shift_Del	Nonsense_Mutation	Nonsense_Mutation	Nonsense_Mutation
SUFU	Splice_Region	Splice_Region	Splice_Region	Splice_Region
ATM	Missense_Mutation	Missense_Mutation	Missense_Mutation	Missense_Mutation
AXIN1	Frame_Shift_Del	Frame_Shift_Del	Frame_Shift_Del	Frame_Shift_Del
AURKA	Missense_Mutation	Missense_Mutation	Missense_Mutation	Missense_Mutation
BCOR		Nonsense_Mutation	Nonsense_Mutation	Nonsense_Mutation
FANCD2	Splice_Site	Splice_Site		
ATR	Frame_Shift_Del		Frame_Shift_Del	
NOTCH1	Frame_Shift_Del Frame_Shift_Ins		Frame_Shift_Del	
RNF43	Frame_Shift_Ins		Frame_Shift_Ins	
NFE2L2	Missense_Mutation			
PMS1	Frame_Shift_Del			
RAD50	Frame_Shift_Del			
MYC	Missense_Mutation			
MRE11	Frame_Shift_Del			
CHEK1	Frame_Shift_Del			
KMT2D	Frame_Shift_Del Frame_Shift_Ins			
BRCA2	Frame_Shift_Del			
NF1	Frame_Shift_Ins			
CDK12	Frame_Shift_Del			
SMARCA4	Frame_Shift_Del×2			
SMARCB1	Splice_Site			
KMT2B	Frame_Shift_Ins Frame_Shift_Del			
BCL11B			Frame_Shift_Del	
CIC			Nonsense_Mutation Frame_Shift_Ins	

## Discussion

This case presents a relatively rare undifferentiated carcinoma of the pancreas with a squamous cell carcinoma component. Genomic analysis identified shared gene mutations between the undifferentiated and squamous cell carcinoma components, suggesting a common origin. The presence of numerous gene mutations in the squamous cell carcinoma component implies the acquisition of distinct mutations, possibly leading to squamous differentiation from the undifferentiated component.

There are several reports on the analysis aimed at elucidating the high malignancy of undifferentiated carcinoma of the pancreas. Naito et al. [8] and Ishida et al. [9] conducted evaluations using immunohistochemical staining with surgical and autopsy specimens. Among undifferentiated carcinoma, a decrease in the expression of epithelial markers and an increase in the expression of mesenchymal markers have been observed in more loosely cohesive sarcomatoid cells, suggesting the importance of epithelial-mesenchymal transition (EMT) in pathogenesis. In this analysis, gene mutations related to EMT, such as KMT2C [10], SUFU [11], and AURKA [12], have been identified, implying their potential importance in the pathogenesis of the present patient.

On the other hand, reports on genome analysis of undifferentiated carcinoma of the pancreas are limited. Luchini et al. focused on undifferentiated carcinoma with osteoclast-like giant cells (UCOGC), noting that the prognosis varies depending on the presence of adenocarcinoma components, and conducted genetic analysis [13]. In this report, whole exon sequencing was performed on six cases of UCOGC, including adenocarcinoma, and teo cases of pure UCOGC. All cases showed mutations in KRAS codon 12, TP53 mutations were also present in seven cases, and other mutations in SMAD4, CDKN2A were also observed. Although mutations in well-known driver genes such as PTEN, BAP1, IDH2, and genes with insufficiently analyzed functions were also found at low frequencies, no specific genetic abnormalities were observed in UCOGC. In a study by Yamamoto et al., genomic analysis of surgical specimens compared adenocarcinoma and undifferentiated carcinoma components, revealing a tendency toward mutations in SWI/SNF complex-related genes (ARID1A, SMARCA2, SMARCA4) in undifferentiated carcinoma [14]. While mutations in KRAS and TP53 were also observed in this case, our study identified mutations in SMARCA4 and SMARCB1 in the squamous cell carcinoma component. This suggests that the components classified as undifferentiated carcinoma, in this case, may be associated with abnormalities in chromatin-related genes. However, it is important to note that morphological anaplasia and abnormalities in chromatin-related genes are not necessarily directly linked.

## Conclusions

In conclusion, we conducted a genomic analysis of pancreatic undifferentiated carcinomas with a squamous cell carcinoma component. This analysis shed light on the tumor's origin and, consistent with previous reports, indicated a correlation with the pathogenesis of EMT. Moreover, it unveiled a potential involvement of chromatin remodeling factor mutations in the morphogenesis of pancreatic undifferentiated carcinoma. Further studies concentrating on the molecular characteristics of pancreatic undifferentiated carcinoma are imperative for a thorough comprehension of its intricate biological behavior.
